# Comparison between Magnification Techniques and Direct Vision in Thyroid Surgery: A Systematic Review and Meta-Analysis

**DOI:** 10.3390/medicina55110725

**Published:** 2019-11-01

**Authors:** Konstantinos Sapalidis, Anastasios Papanastasiou, Varvara Fyntanidou, Zoi Aidoni, Nikolaos Michalopoulos, Athanasios Katsaounis, Aikaterini Amaniti, Paul Zarogoulidis, Charilaos Koulouris, Dimitrios Giannakidis, Aris Ioannidis, Iason-Nikolaos Katsios, Konstantinos Romanidis, Panagoula Oikonomou, Isaak Kesisoglou, Christoforos Kosmidis

**Affiliations:** 1 3rd Department of Surgery, “AHEPA” University Hospital, Aristotle University of Thessaloniki, Medical School, 541 24 Thessaloniki, Greece; tasos-papana@hotmail.com (A.P.); bfyntan@yahoo.com (V.F.); zoiaidoni@yahoo.com (Z.A.); nmichalopoulos1@outlook.com (N.M.); athanasios_katsaounis@hotmail.com (A.K.); pzarog@hotmail.com (P.Z.); charilaoskoulouris@gmail.com (C.K.); giannakidis.d@gmail.com (D.G.); Konstantinossapalidis2@outlook.com (A.I.); tpapavramidis@outlook.com (I.-N.K.); isaackesisoglou@outlook.com (I.K.); dr.ckosmidis@gmail.com (C.K.); 2 Anesthisiology Department, “AHEPA” University Hospital, Aristotle University of Thessaloniki, Medical School, 541 24 Thessaloniki, Greece; amanitik@gmail.com; 3 Second Department of Surgery, University Hospital of Alexandroupolis, Medical School, Democritus University of Thrace, 68100 Alexandroupolis, Greece; romanidis@yahoo.com (K.R.); Pen.ek@hotmail.com (P.O.)

**Keywords:** thyroid surgery, hypocalcemia, laryngeal nerve, meta-analysis

## Abstract

*Background and Objectives*: The most common complications after conventional thyroid surgery in adult patients are recurrent laryngeal nerve (RLN) injury and hypocalcemia. Magnification techniques (surgical loupes or surgical microscope) are used for identification of RLN and parathyroid glands to diminish these complications although more evidence is necessary to assess their safety and efficacy in comparison with direct vision. *Methods and Materials*: Electronic databases (Pubmed, Cochrane Library, Scopus) as well as gray literature sources were searched for randomized controlled trials (RCTs) comparing the frequency of transient/permanent RLN injury and hypocalcemia after thyroid surgery by using magnification techniques and direct vision for identification of RLN and parathyroid glands until October 17, 2019. The main outcomes were transient/permanent RLN injury and hypocalcemia. For all outcomes, 95% confidence intervals (95% CI) were used. Statistical analysis was performed with RevMan 5.3. *Results*: Systematic review and meta-analysis included 3 RCTs with 437 patients overall. Magnification techniques did not significantly affect the risk of occurrence of transient RLN injury (OR = 0.38, 95% CI (0.11–1.35), I^2^ = 0%) and transient hypocalcemia (OR = 0.31, 95% CI (0.09–1.09), I^2^ = 23%) compared with direct vision. Included RCTs demonstrated only two patients with permanent hypocalcemia and another one with permanent RLN injury, who belonged to the direct vision group. *Conclusion*: The use of magnification techniques for identification of RLN and parathyroid glands seems to be as safe as direct vision. However, they do not decrease the risk of RLN injury and transient hypocalcemia after thyroid surgery compared with direct vision. Finally, further prospective research should be conducted as the sample among the studies was small.

## 1. Introduction

Total thyroidectomy and unilateral thyroid lobectomy are common surgical procedures. However, they can lead to specific complications. The most common of them are related to laryngeal nerves (recurrent laryngeal nerve (RLN), external branch of superior laryngeal nerve (EBSLN)) and parathyroid glands [1]. Two important risk factors of these complications are the extent of thyroidectomy and the surgeon’s experience [2].

Therefore, a microsurgical approach in thyroid surgery by using magnifying loupes or surgical microscope is believed to enhance the surgeon’s precision. Also, it was stated that the above anatomical structures could be identified in an easier way during the operation [3]. The first microsurgical thyroidectomy was reported in 1975 by Atti et al. [4]. Since then, the experience of some expertized centers in microsurgical approach of thyroidectomy have been presented in the literature. According to them, the surgical microscope is recommended in thyroid surgery due to a lower incidence of complications [5,6,7,8,9,10]. However, the only prospective controlled study that has been reported is the one by Seven et al. in 2004 [11].

The current systematic review and meta-analysis aimed to investigate if the use of magnification techniques in thyroid surgery is associated with the risk of RLN injury and hypocalcemia compared with direct vision. Such an association would have obvious clinical implications for safety and efficacy of magnification techniques in thyroid surgery.

## 2. Materials and Methods

Randomized controlled trials (RCTs) comparing the frequency of specific complications after thyroid surgery (total thyroidectomy or unilateral lobectomy), due to either benign or malignant disease, by using magnification techniques (surgical microscope or loupe magnification) or direct vision to identify the RLN and the parathyroid glands were searched for in the PubMed, Cochrane Library, and Scopus databases as well as “gray literature” sources (opengrey, greynet) until October 17, 2019. No patients had any previous neck surgery and no restriction was set about central neck dissection (CND). RCTs with minimally invasive video-assisted thyroidectomy (MIVAT) were excluded. Observational studies were not included in the meta-analysis.

The literature was reviewed by two researchers (P.A., M.S.). Any discrepancies between them were resolved in agreement with a third researcher (S.K.). The terms “thyroid”, magnification”, “microsurgery”, “microscope”, “loupes”, and “thyroidectomy” were combined in the search strings. The outcomes examined were transient hypocalcemia, permanent hypocalcemia, transient RLN injury, and permanent RLN injury. Transient hypocalcemia appears with a variety of definitions in the literature. Studies with comparable definitions of transient hypocalcemia were selected. Regarding the transient and permanent RLN injury, the number of nerves at risk was calculated depending on whether lobectomy (potential unilateral RLN injury) or total thyroidectomy (potential bilateral RLN injury) was performed. The status of the vocal cords of all patients was checked postoperatively by laryngoscope to define potential injury. RLN was identified by any means.

Two researchers proceeded to data extraction. Data were deployed for statistical analysis using Revman 5.3. (Cochrane Community, New York, NY, USA). The random effects model was preferred for the meta-analysis. The results are presented as Odds Ratio (OR) with confidence intervals (CI). Additionally, I^2^ was demonstrated in every forest plot to assess the degree of heterogeneity. The risk of bias of the included studies in the meta-analysis was estimated through the RoB 2 tool (version 2018) [12].

## 3. Results

**Figure 1:**
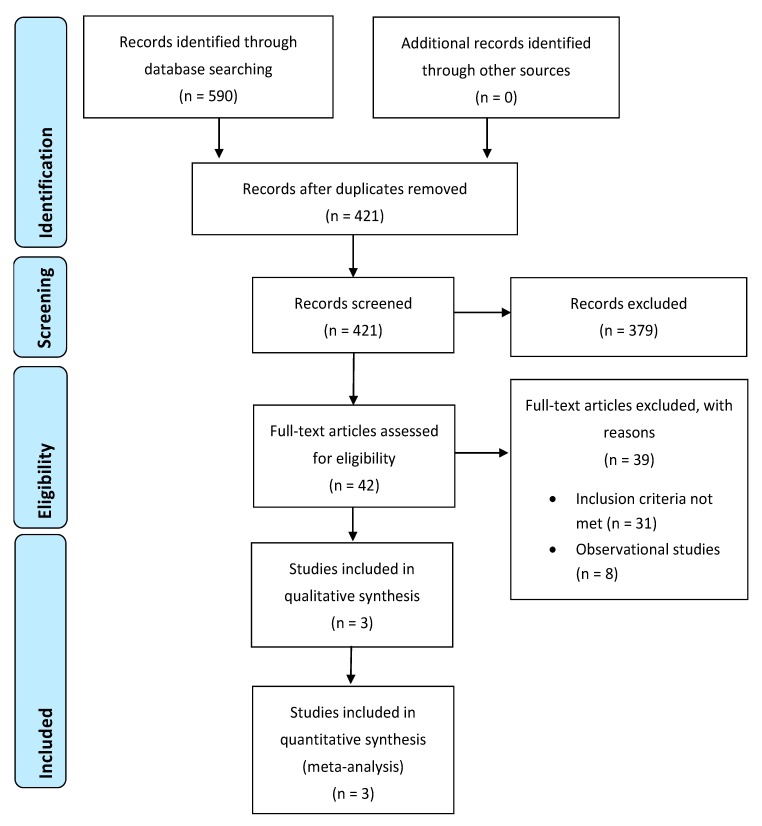
Flow diagram of the study selection process.

Initially, 196 studies were found in Pubmed, 40 in Cochrane library, 354 in Scopus, and 0 in gray literature. After duplicates were removed, 421 studies remained. These were reviewed by the researchers in two stages. Firstly, the title and abstract were checked and then the full text. Finally, three studies were included in the meta-analysis with 437 adult patients (Figure 1). The basic characteristics of the selected studies are presented in Table 1.

However, in their study, Seven et al. included patients that had undergone either total thyroidectomy or unilateral lobectomy where the surgical microscope (4×−10×) was used [11], while the others included only patients with total thyroidectomy where loupe magnification (2.5×) was used [13,14]. The definition of temporary hypocalcemia was not the same for any study, but these were comparable. A transplant of a parathyroid gland was reported only in three cases by Testini et al. [13]. Neuromonitoring was not implemented in any study for RLN identification.

**Figure 2:**
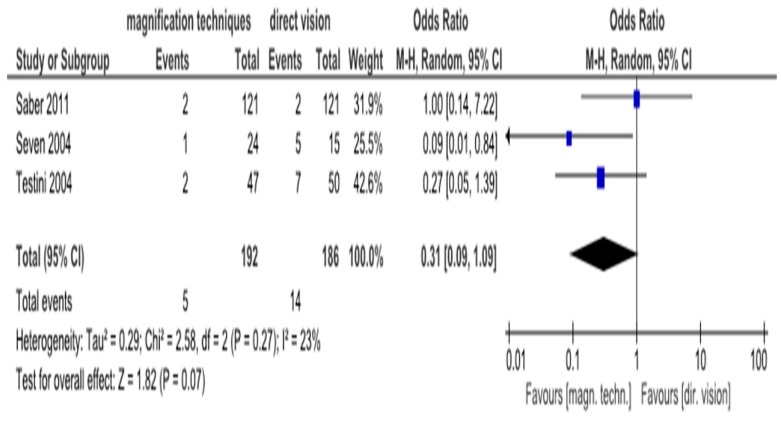
Forest plot comparing identification of parathyroid glands by magnification techniques and direct vision on transient hypocalcemia.

The meta-analysis of the three RCTs did not show a significant effect of magnification techniques on transient hypocalcemia compared to direct vision (OR = 0.31, 95% CI (0.09–1.09), I^2^ = 23%) (Figure 2).

**Figure 3:**
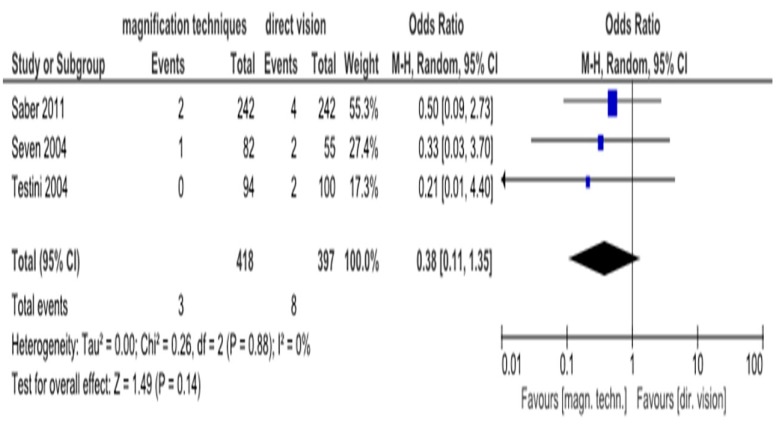
Forest plot comparing identification of RLN by magnification techniques and direct vision on transient RLN injury.

Furthermore, although more patients from the direct vision group suffered from transient RLN injury, there was no statistically significant difference compared to the control group (OR = 0.38, 95% CI (0.11–1.35), I^2^ = 0%) (Figure 3).

Included RCTs demonstrated only two patients with permanent hypocalcemia and only one with permanent RLN injury. Therefore, it was decided not to perform meta-analysis for these complications. These patients belonged to the direct vision group.

The study by Testini et al. [13] was judged to be at high risk of bias due to the fact that there was no clear evidence for the implementation of allocation concealment. A detailed presentation of risk of bias assessment of both studies is given in Table 2. In addition, publication risk of bias assessment was considered not applicable because only three studies were included in the meta-analysis. Therefore, there are no funnel plots demonstrated in the current study.

## 4. Discussion

The purpose of this systematic review and meta-analysis was to investigate the impact of the magnification techniques (surgical microscope or loupe magnification) on identification of parathyroid glands and RLN in thyroid surgery, compared to direct vision. The study concluded that the use of magnification techniques has no statistically significant effect on the risk of transient hypocalcemia and transient RLN injury compared with direct vision. The heterogeneity was (I^2^ = 23%) and (I^2^ = 0%), respectively, and was not judged as high. Heterogeneity of temporary hypocalcemia is believed to derive from the different definitions of it used in the included studies. There was not enough data to proceed to statistical analysis of the permanent hypocalcemia and RLN injury. Three patients from the direct vision group suffered from the above complications.

These results are not in total compliance with the study by Pata et al., in which the use of loupe magnification (2.5×) seemed to decrease the rate of inadvertent parathyroid glands removal and transient hypocalcemia (biochemical and clinical) but had no effect on the RLN injury. This study was excluded from the current meta-analysis because the control data was retrospective [15]. It is worth noting that six more studies were found through the review of the literature which present the experience of some expertized centers in microsurgical approach of thyroidectomy. All these studies recommend the routine use of magnification techniques in thyroid surgery [5,6,7,8,9,10]. In addition, Davidson et al. compared the surgical microscope with surgical loupes and conjectured that there were no significant differences between the two devices regarding the complications of the operation. However, the use of the surgical microscope may benefit the surgeons experiencing neck pain symptoms or other occupationally related musculoskeletal disorders [16].

A former systematic review and meta-analysis were conducted in 2015 of the effect of various surgical measures on hypocalcemia following bilateral thyroidectomy. One of them was the use of magnification loupes, but only the study by Testini et al. was included. Therefore, it was stated by Antakia et al. that the utilization of loupes was not preventative for hypocalcemia, despite the fact that the RCT study by Saber et al. and observational studies, such as the one by Pata et al., were not taken into account [17]. The main difference is that in the present study, all the magnification techniques that could be applied in conventional thyroid surgery were combined. Moreover, the literature search was more precise, because there was only one specific intervention under investigation. Finally, in the current systematic review, more outcomes were set primarily concerning the most common complications of thyroid surgery.

Regarding the limitations of the study, there were very few appropriate studies in the literature for the current topic. Initially, 421 studies were found through the literature search. Among them, 379 were excluded because they were irrelevant at the screening of title and abstract. Subsequently, 42 studies were screened at a full-text level. Most of them were observational, while only three were RCTs and were included eventually in the meta-analysis. However, different methods and degrees of magnification were applied in them (Testini et al., Saber et al.: loupe magnification 2.5×, Seven et al.: surgical microscope 4×−10×). Moreover, no clear reference existed about the allocation concealment in Testinis’s and Seven’s RCTs. In addition, RCTs were screened that compared MIVAT with conventional thyroidectomy in the studies by Miccoli et al., Lombardi et al., and El Labban et al. [18,19,20]. These studies were excluded because in MIVAT, the identification of both RLN and parathyroid glands is performed endoscopically. Furthermore, a systematic review and meta-analysis comparing MIVAT with conventional thyroidectomy had already been performed in 2013 that showed no difference in the incidence of hypocalcemia and RLN injury [21].

The most common complications after thyroid surgery are RLN injury and hypocalcemia. An excellent anatomical knowledge is necessary for the surgeon to identify and preserve every parathyroid gland and RLN [13]. Moreover, the surgical techniques must be very vigilant, especially when patients undergo a total thyroidectomy with CND. Bilateral CND includes the removal of prelaryngeal, pretracheal, and both the right and the left paratracheal nodal basins. Homolateral CND includes removal of the prelaryngeal, pretracheal, and paratracheal nodal basins on the side of the tumor. Hence, these patients are at higher risk for hypocalcemia than those who undergo only total thyroidectomy [22,23].

## 5. Conclusions

In conclusion, the use of magnification techniques (surgical microscope and loupe magnification) for the identification of parathyroid glands and RLN has no effect on the risk of temporary hypocalcemia and transient RLN injury after thyroid surgery. In addition, they appeared to be as safe as direct vision. On the contrary, in the recent American Thyroid Association (ATA) statement on postoperative hypoparathyroidism, it was claimed that the use of surgical loupes could decrease the risk of the specific complication [2]. Concerning permanent hypocalcemia and RLN injury, the data were inadequate to proceed to meta-analysis. Therefore, more RCTs are necessary to be conducted to form a more integrated aspect of the impact of magnification techniques on the specific complications of thyroid surgery. In addition, magnification techniques could be combined with intraoperative neuromonitoring (IONM) regarding the identification of laryngeal nerves [24]. However, a steep learning curve is necessary for IONM implementation. Otherwise, the risk of RLN injury may be increased [25]. Finally, because thyroid surgery and magnification techniques require expertise, the surgeon’s experience is a risk factor that should always be taken seriously into account [26].

## Figures and Tables

**Table 1:** Basic characteristics of the included studies.

First Author	Year	Country	Direct Vision Group	Magnification Technique Group	Type of Surgery	Magnification Technique	Definition of Transient Hypocalcemia
Seven	2004	Turkey	40	58	Total thyroidectomy (39) or unilateral lobectomy (59)	Surgical microscope (4×−10×)	Patient with symptoms or an ionized calcium value of less than 1.0 mmol/L
Testini	2004	Italy	50	47	Total thyroidectomy	Loupe magnification (2.5×)	Calcemia lower than 10% of the preoperative value, or symptoms of hypocalcemia
Saber	2011	Egypt	121	121	Total thyroidectomy	Simple binocular loupe (2.5×)	Calcium level lower than 8.0 mg/dL in at least two consecutive samples (twice daily for three days)

**Table 2:** Summary of risk of bias assessment for the studies included in the meta-analysis.

Study	Year	Randomization Process	Deviations from Intended Interventions	Missing Outcome Data	Measurement of the Outcome	Selection of the Reported Result	Overall Risk of Bias
Seven [11]	2004	Some Concerns	Low Risk	Low Risk	Low Risk	Low Risk	Some Concerns
Testini [13]	2004	High Risk	Low Risk	Low Risk	LowRisk	Low Risk	High Risk
Saber [14]	2011	Low Risk	Low Risk	Low Risk	Low Risk	Low Risk	Low Risk

Overall risk of bias judgment. Low Risk: The study is judged to be at low risk of bias for all domains for this result. Some Concerns: The study is judged to raise some concerns in at least one domain for this result, but not to be at high risk of bias for any domain. High Risk: The study is judged to be at high risk of bias in at least one domain for this result OR the study is judged to have some concerns for multiple domains in a way that substantially lowers confidence in the result [12].
